# Whole-Genome Sequences of Seven Listeria monocytogenes Strains from Different Stages of a Poultry Meat Production Chain

**DOI:** 10.1128/MRA.00088-19

**Published:** 2019-03-14

**Authors:** Victoria López-Alonso, Sagrario Ortiz, Joaquín V. Martínez-Suárez

**Affiliations:** a Unidad de Biología Computacional, UFIEC, Instituto de Salud Carlos III, Majadahonda, Madrid, Spain; b Departamento de Tecnología de Alimentos, Instituto Nacional de Investigación y Tecnología Agraria y Alimentaria (INIA), Madrid, Spain; University of Southern California

## Abstract

Here, we present the draft genome sequences of seven Listeria monocytogenes strains isolated during three independent studies carried out in three stages of a poultry meat production chain. The genome sequences of these strains obtained from different stages can help to understand the possible transmission of L. monocytogenes.

## ANNOUNCEMENT

Listeriosis is a foodborne disease caused by the bacterium Listeria monocytogenes. The disease has a high mortality rate, especially among newborns, the elderly, and immunocompromised adults (1). L. monocytogenes is very common in food production environments, mainly food of animal origin, and usually colonizes chicken abattoirs and chicken processing plants (2). Consequently, L. monocytogenes is also frequently found in raw chicken and other poultry meat in retail stores (3). There are few studies on the transmission of L. monocytogenes strains along the chicken meat production and supply chain. Herein, this type of study is especially important to define the factors involved in such transmission.

The objective of this study was to characterize the genomes of a selected group of L. monocytogenes strains present during several stages of a chicken meat production and supply chain (abattoir, processing plant, and retail). The origin of these strains, as well as their molecular subtypes, were previously published in three independent articles (4–6). Knowledge about the sources of contamination of chicken meat in retail would help to improve the risk assessment and implementation of preventive measures.

Four isolates corresponding to strains obtained from the abattoir (A3, A7, A10, and A13) were sequenced by whole-genome sequencing (WGS). Two additional isolates from the processing plant (P12 and P17), together with one isolate from the retail stage (R6), were also subjected to WGS for comparative purposes (Table 1) (4–6; our unpublished data). The seven isolates were grown in tryptic soy-yeast extract broth at 37°C, and genomic DNA was extracted using a bacterial genomic DNA purification kit (Wizard; Promega, Madison, WI, USA) according to the manufacturer’s protocol.

**TABLE 1:** Characteristics and accession numbers of genomes of the seven L. monocytogenes strains

Characteristic^*a*^	Data for strain:						
	A3	A7	A10	A13	P12	P17	R6
Original strain name	CAL190404_14 a	CAL150304_15 a	CAL240504_38 a	CAL090505_12	CAL250805_7 a	CAL191004_2c	H180405_63 b
Serotype	1/2a	1/2a	1/2a	1/2a	1/2a	1/2a	1/2a
MLST	ST121	ST31	ST121	ST31	ST31	ST3	ST31
Origin	Abbatoir	Abbatoir	Abbatoir	Abbatoir	Processing plant	Processing plant	Retail
GenBank accession no.	RXOV00000000	RXOS00000000	RXOP00000000	RXOR00000000	RXOQ00000000	RXOU00000000	RXOT00000000
BioSample no.	SAMN10584524	SAMN10584527	SAMN10584530	SAMN10584528	SAMN10584529	SAMN10584525	SAMN10584526
SRA accession no.	SRX5164292	SRX5164288	SRX5164291	SRX5164289	SRX5164290	SRX5164293	SRX5164294
No. of reads for assembly	4,504,630	4,031,884	6,169,012	7,842,256	7,799,488	2,463,762	4,564,458
Genome size (bp)	3,105,826	3,088,362	3,111,546	3,112,968	3,053,254	2,982,329	3,056,197
No. of contigs	37	40	54	60	36	27	48
*N*_50_ value (bp)	465,517	473,937	482,166	473,938	584,633	518,569	584,755
G+C content (%)	37.9	38	37.9	37.82	38	38.1	38.1
Total no. of genes	3,221	3,174	3,226	3,224	3,137	3,026	3,149
Total no. of CDS	3,153	3,040	3,116	3,154	3,064	2,958	3,097
No. of RNA genes	68	70	68	70	73	68	70
No. of tRNAs	56	56	56	56	56	56	56
No. of complete rRNAs (types)	3, 1, 1 (5S, 16S, and 23S)	3, 1 (5S and 23S)	3, 1, 1 (5S, 16S, and 23S)	4, 1 (5S and 23S)	3, 1 (5S and 23S)	3, 1, 1 (5S, 16S, and 23S)	2, 1 (5S and 23S)
No. of ncRNAs	4	4	4	4	4	4	4
No. of proteins	3,116	3,040	3,116	3,075	2,993	2,926	3,011
CRISPR arrays	3	2	3	2	2	2	2

a ST, sequence type; CDS, coding DNA sequences; ncRNAs, noncoding RNAs.

Library preparation was carried out using the TruSeq technology (Illumina, San Diego, CA, USA), and a 2 × 250-nucleotide multiplexed paired-end sequencing run was performed using the MiSeq platform (Illumina). Prior to sequence assembly, the quality of the sequences was assessed by FastQC v 0.11.7 (https://www.bioinformatics.babraham.ac.uk/projects/fastqc/). Trimming of the adapter sequences and low-quality bases (<Q20) from raw reads was performed using Trim Galore v 0.5.0 (https://www.bioinformatics.babraham.ac.uk/projects/trim_galore/). The sequences were assembled separately using the *de novo* assembler SPAdes v 3.12.0 (7) with activation of the “–careful” option, and raw assemblies were filtered for size larger than 500 bp and coverage of more than 25-fold. The NCBI Prokaryotic Genome Annotation Pipeline (PGAP) v 4.7 was used for gene prediction (8).

*In silico* multilocus sequence typing (MLST) analysis of the genome of each strain was conducted using MLST 2.0 (9). The prophage sequences in each genome were identified using the PHAge Search Tool Enhanced Release (PHASTER) Web server (2016) (10).

A description of the characteristics of the seven L. monocytogenes strains and their genomes is presented in Table 1. The information obtained through WGS can be valuable for the identification of sequence differences that exist among these strains and help to understand why these strains have revealed such different transmission potentials.

### Data availability.

The draft genome sequences reported here have been deposited in DDBJ/ENA/GenBank under the BioProject number PRJNA509563 and under the individual accession numbers listed in Table 1. The versions described in this paper are the ﬁrst versions.
